# Subjective cognitive complaints in patients with progressive supranuclear palsy

**DOI:** 10.3389/fneur.2023.1326571

**Published:** 2023-12-14

**Authors:** Jun Seok Lee, Jong Hyeon Ahn, Jong Mok Ha, Jinyoung Youn, Jin Whan Cho

**Affiliations:** ^1^Department of Neurology, Samsung Medical Centre, Sungkyunkwan University School of Medicine, Seoul, Republic of Korea; ^2^Neuroscience Centre, Samsung Medical Centre, Seoul, Republic of Korea

**Keywords:** progressive supranuclear palsy, subjective cognitive complaints, cognitive impairment, Parkinson’s disease, mild cognitive impairment

## Abstract

**Introduction:**

Subjective cognitive complaints (SCC) refer to self-reported cognitive decline that may or may not be reflected in objective neuropsychological evaluations. Such SCC are prevalent in neurodegenerative diseases, including Parkinson’s disease (PD), but the prevalence and clinical features in patients with progressive supranuclear palsy (PSP) have not been investigated.

**Methods:**

We recruited 83 PSP patients without dementia and investigated their SCC using a semi-structured interview. Comprehensive neuropsychological test results and patient clinical features were compared according to presence of SCC and underlying cognitive state.

**Results:**

Among the 83 patients, 16 had normal cognition (NC), 67 had mild cognitive impairment (MCI), and 36 (43.4%) reported SCC. Among NC patients, 37.5% (6/16) had SCC, while 44.8% (30/67) of MCI patients reported SCC. There were no differences between the neuropsychological test results or demographic and clinical characteristics of PSP patients with or without SCC in the NC group. The demographic and clinical characteristics of the MCI+SCC (MCI with SCC)and MCI-SCC (MCI without SCC) groups were comparable, but the MCI+SCC group had significantly worse neuropsychological scores than the MCI-SCC group, particularly in tests assessing attention, language, visual memory, and fronto-executive function domains.

**Discussion:**

While SCC are commonly reported by PSP patients, patients with PSP and MCI+SCC had worse cognitive function than those who did not report SCC. These findings suggest that SCC in PSP patients with MCI could be a worsening sign of cognitive function. Therefore, it is crucial for physicians to assess SCC in PSP patients and to provide timely diagnosis and management of cognitive decline.

## Introduction

Cognitive impairment is a common non-motor symptom among patients with progressive supranuclear palsy (PSP) (1). Executive function is the most commonly affected cognitive domain in PSP, but other cognitive domains, including memory, language, attention, and visuospatial function, may also be affected (2–4). Patients with PSP exhibit a broad spectrum of cognitive decline, ranging from normal cognition (NC) to severe dementia (5). In the early stages of the disease, mild cognitive impairment (MCI) is the most common type of cognitive dysfunction with a prevalence of 43%, followed by dementia (41%) and NC (16%) (5). The majority of PSP patients progresses to dementia over time, with an incidence rate of 241 per 1,000 patients/year (5). The presence of MCI at the baseline neuropsychological evaluation of PSP patients has been identified as a predictive factor for development of dementia, while no significant differences have been observed in motor symptoms or demographic characteristics between those who developed dementia and those who did not (5). However, the predictive value of subjectively recognised symptoms has not been investigated.

Subjective cognitive complaints (SCC) refer to self-reported experiences of cognitive decline that may or may not be reflected in objective neuropsychological test results (6). Patients with Parkinson’s disease (PD) frequently report SCC, with an overall prevalence of 36.3% among cognitively normal PD patients, and a range of 6.3% to 82.9% depending on the study (7). Among PD patients, those with SCC have been reported to display several interesting clinical features not seen in those without SCC. Chua et al. (8) found that the implications of SCC in PD patients can differ based on cognitive status, with SCC in PD suggesting the presence of an underlying affective disorder in patients with NC but not in those with MCI. In PD patients with MCI, those with SCC exhibit more severe cognitive impairment in the objective cognitive tests and higher non-motor burden than those who do not have SCC (9–11). In longitudinal studies, the presence of SCC in PD patients at baseline has been associated with greater risk of progressing from NC to MCI (11–13) and then to dementia (14). Since the greater prevalence and severity of cognitive decline in PSP than PD have been reported, the investigation of the prevalence and clinical features of PSP patients with SCC is also important (15). In the present study, we evaluated the prevalence of SCC in patients with PSP as well as the clinical features and objective cognitive function based on cognitive status.

## Methods

### Ethical approval

All the methods and experiments were designed and performed in accordance with the Declaration of Helsinki and relevant guidelines and regulations provided by the policies of Nature Portfolio journals. This research was reviewed and approved by the Institutional Review Board of Samsung Medical Centre (IRB No. SMC-SMC 2023-03-123), and all enrolled subjects provided written informed consent.

### Participants

In this cross-sectional study, eligible participants with probable PSP were identified at the Movement Disorders Clinic of Samsung Medical Centre, from April 2015 to August 2022. The diagnosis of PSP was based on the Movement Disorders Society (MDS) clinical criteria for PSP, and the subtypes of PSP were assigned using clinical features. In this study, we chose to use the term SCC instead of other terms such as “subjective cognitive decline” (SCD) or “subjective memory complaints” (SMC) to provide clarity in our terminology (7). The presence of SCC was established through a semi-structured interview (Supplementary Table S1) conducted on the same day as the neuropsychological battery. We included only PSP patients who had completed the neuropsychological battery within 36 months from the onset of the disease, as the primary objective of this study was to investigate the clinical impact of SCC in the early stages of the disease (16).

We assessed the demographics and clinical features of patients including age, sex, disease duration, year of education, unified Parkinson’s disease rating scale (UPDRS) III score (17), and Hoehn and Yahr (H&Y) stage (18). The Korean version of the Mini Mental State Examination (K-MMSE) (19), Geriatric Depression Scale (GDS) (20), and Neuropsychiatric Inventory (NPI) (21) were used to assess general cognitive function, depressive symptoms, and neuropsychiatric symptoms, respectively, in the 83 patients. The diagnosis of MCI was based on the criteria proposed by the MDS (22). For the analyses, we excluded patients with dementia (23), as we wanted to focus on the significance of SCC during the pre-dementia stage.

### Neuropsychological tests

We assessed attention using the digit span forward, digit span backward, and the trail-making test type A (TMT-A) and type B (TMT-B). Language function was evaluated with the Korean version of the Boston naming test (K-BNT). Visuospatial function was assessed through the copying task of the Rey Osterrieth complex figure test (RCFT). When testing patients’ visual memory, the neuropsychology group checked if the patients really saw the imaging. If they failed at an initial try, the neuropsychology group gave them several chances to see the imaging until ensuring their visual memory would be ready to be tested. Memory was evaluated using the 20 min delayed recall score within the Seoul verbal learning test (SVLT) and RCFT. Executive function was measured using the Stroop colour reading task and semantic fluency tasks, including animals, supermarket, and phonemes, using the controlled oral word association test (COWAT) (16).

### Statistical analyses

All data are presented as mean ± standard deviation. Data were examined for normality using the Kolmogorov–Smirnov test. The patients were classified as having NC or MCI based on the results of the neuropsychological tests. The PSP patients with NC and MCI were further divided into subgroups with SCC (NC + SCC and MCI + SCC) or without SCC (NC − SCC and MCI − SCC). Group comparisons of baseline data were performed using the chi-square or student’s *t*-test depending on the variable. All tests were two-tailed, and the α level was set at *p* < 0.05. All statistical analyses were performed using SPSS (version 28.0; IBM Inc., United States) software for Windows.

## Results

### Participants

**Figure 1:**
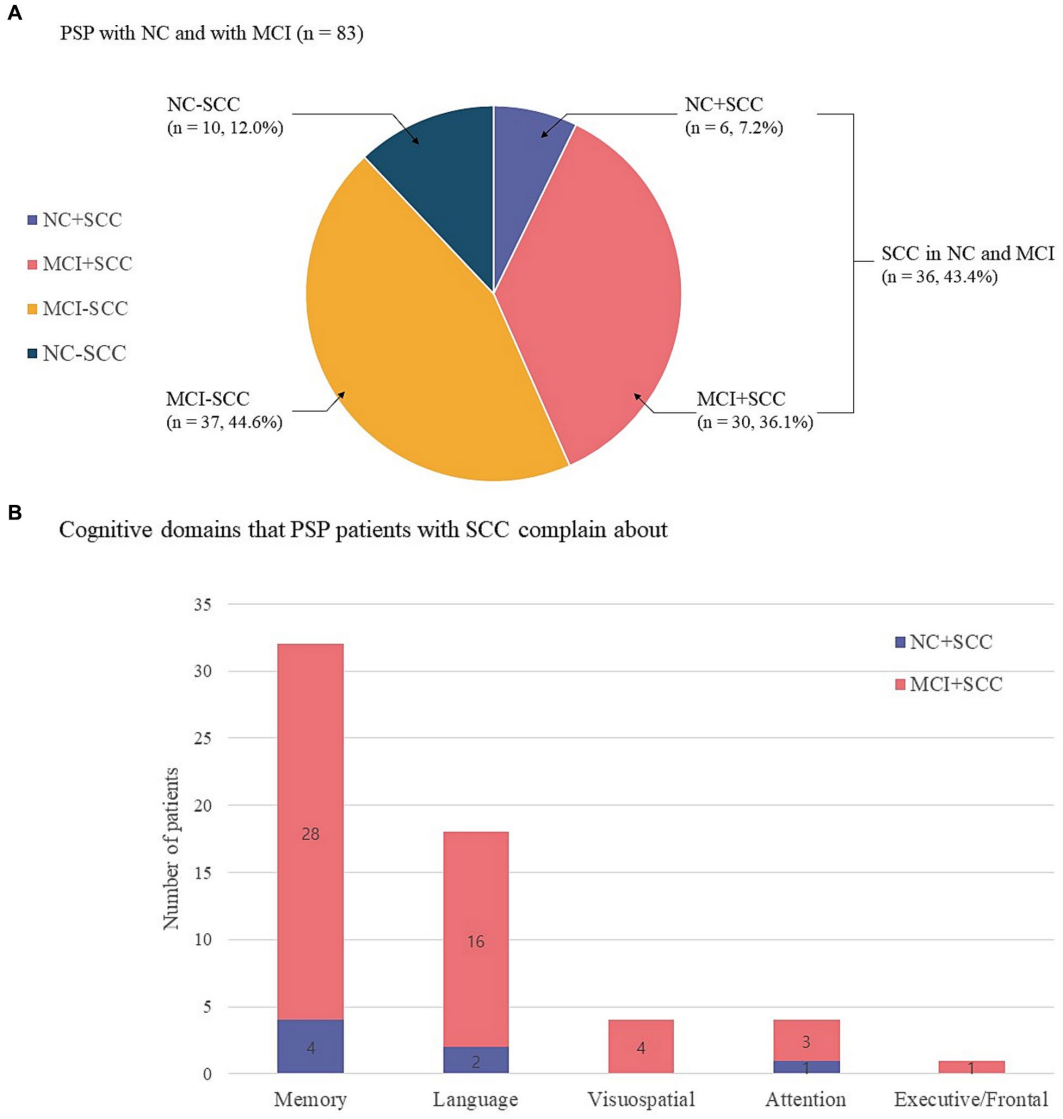
Sixteen of 83 progressive supranuclear palsy patients were classified as having normal cognition (NC), and 67 had mild cognitive impairment (MCI). The prevalence of subjective cognitive complaints (SCC) was 43.4% (36/83) regardless of the underlying cognitive state. The prevalence of NC with SCC (NC + SCC), NC without SCC (NC − SCC), MCI with SCC (MCI + SCC), and MCI without SCC (MCI − SCC) was 7.2%, 12.0%, 36.1%, and 44.6%, respectively **(A)**. Among progressive supranuclear palsy (PSP) patients with normal cognition and subjective cognitive complaints (NC + SCC), memory complaints were most frequent (*n* = 4), followed by alteration of language (*n* = 2) and attention (*n* = 1) domains. Among PSP patients with mild cognitive impairment and subjective cognitive impairment (MCI + SCC), memory complaints were most frequent (*n* = 28), followed by alteration of language (*n* = 16), visuospatial (*n* = 4), executive/frontal (*n* = 3), and attention (*n* = 1) cognitive domains. Of the 36 patients who reported SCC, 17 had complaints in one cognitive domain, 15 had complaints in two domains, and 4 PSP patients reported complaints in three cognitive domains **(B)**.

The present study included 83 patients with progressive supranuclear palsy (PSP). Of these, 65 (78.3%) also had Richardson’s syndrome (PSP-RS), 13 (15.7%) had predominant parkinsonism (PSP-P), and 5 (6.0%) had progressive gait freezing (PSP-PGF). Sixteen of 83 patients were classified into the NC group and 67 into the MCI group. The prevalence of SCC was 43.4% (36/83) regardless of the underlying cognitive state (Figure 1A). The prevalence of NC + SCC, NC − SCC, MCI + SCC, and MCI − SCC was 7.2%, 12.0%, 36.1%, and 44.6%, respectively (Table 1). When considering only patients with NC, the prevalence of SCC was 37.5% (6/16), comparable to the prevalence of SCC (43.4%, 30/67) in PSP patients with MCI (*p* = 0.598).

**Table 1:** Comparing of clinical and neuropsychological tests depending on the subjective cognitive complaints in progressive supranuclear palsy patients with normal cognition and mild cognitive impairment.

	NC (*n* = 16, 19.3%)		*p*-value	MCI (*n* = 67, 80.7%)		*p*-value
	NC + SCC (*n* = 6, 7.2%)	NC – SCC (*n* = 10, 12.0%)		MCI + SCC (*n* = 30, 36.1%)	MCI − SCC (*n* = 37, 44.6%)	
Age, years	70.2 ± 4.6	71.5 ± 7.8	0.512	72.8 ± 6.3	69.3 ± 9.0	0.073
Sex, male (%)	4 (66.7)	6 (60.0)	0.608	15 (50.0)	23 (62.2)	0.335
Disease duration, months	16.7 ± 10.2	17.0 ± 10.8	0.952	16.7 ± 10.9	17.0 ± 10.1	0.910
Education, years	10.3 ± 4.0	12.6 ± 3.4	0.248	9.7 ± 5.1	10.9 ± 4.1	0.278
Predominance type (RS/P/PGF, n)	4/0/2	8/1/1	0.354	27/0/3	26/4/7	0.085
UPDRS III	23.8 ± 8.6	23.9 ± 16.5	0.994	28.7 ± 13.9	29.7 ± 16.7	0.806
H&Y stage	2.7 ± 0.5	2.9 ± 0.5	0.332	3.1 ± 0.6	3.1 ± 0.6	0.942
MMSE	25.5 ± 0.7	24.5 ± 4.5	0.783	22.7 ± 3.1	21.9 ± 4.3	0.552
GDS	6.5 ± 5.2	5.7 ± 5.0	0.764	7.7 ± 4.3	7.0 ± 4.0	0.541
NPI	8.6 ± 5.2	6.2 ± 9.5	0.140	7.6 ± 7.6	4.7 ± 6.4	0.339

PSP, progressive supranuclear palsy; NC, normal cognition; MCI, mild cognitive impairment; SCC, subjective cognitive complaints; RS, Richardson’s syndrome; P, with predominant parkinsonism; PGF, with progressive gait freezing; UPDRS, Unified Parkinson’s disease rating scale; H&Y stage, Hoehn and Yahr stage; GDS, geriatric depression scale; NPI, neuropsychiatric inventory.

The altered cognitive domain most frequently reported by the NC + SCC group was that for memory (*n* = 4), followed by those for language (*n* = 2) and attention (*n* = 1). In the MCI + SCC group, patient complaints most frequently addressed alterations in memory (*n* = 28), followed by language (*n* = 16), visuospatial (*n* = 4), executive/frontal (*n* = 3), and attention (*n* = 1) domains (Figure 1B). Among the 36 patients who had SCC, 17 had complaints in one cognitive domain, 15 patients had complaints in two domains, and four PSP patients reported complaints in three cognitive domains.

### Comparisons of clinical and neuropsychological tests depending on subjective cognitive complaints in PSP patients with NC or MCI

Sixteen patients were classified as having NC, and there were no statistically significant differences between the demographic and clinical characteristics of the NC + SCC and NC − SCC groups (Table 1). Additionally, no significant differences were found in the neuropsychological test results between the two groups (Table 2). A total of 67 patients was diagnosed with MCI, and 30 of them were diagnosed with MCI + SCC (36.1%). The demographic and clinical characteristics of the MCI + SCC and MCI − SCC groups were comparative (Table 1). The MCI + SCC group had significantly worse scores in neuropsychological tests addressing attention (digit span forward and backward), language (K-BNT), visual memory (RCFT immediate recall and RCFT delayed recall), and fronto-executive function (COWAT-animal and COWAT-supermarket) than those without SCC (Table 2).

**Table 2:** Comparison of neuropsychiatric tests according to the presence of subjective cognitive complaints in progressive supranuclear palsy.

	NC (*n* = 16, 19.3%)		*p*-value	MCI (*n* = 67, 80.7%)		*p*-value
	NC + SCC (*n* = 6, 7.2%)	NC − SCC (*n* = 10, 12.0%)		MCI + SCC (*n* = 30, 36.1%)	MCI − SCC (*n* = 37, 44.6%)	
Digit span forward	6.2 ± 1.3	6.1 ± 1.3	0.694	5.2 ± 1.3	6.2 ± 1.3	**0.004**
Digit span backward	3.5 ± 0.5	4.4 ± 1.6	0.221	2.7 ± 1	3.5 ± 1.0	**0.003**
TMT-A	32.8 ± 8.8	29.9 ± 16.5	0.356	53.9 ± 42.1	41.5 ± 28.3	0.156
TMT-B	58.7 ± 22.5	58.8 ± 39.6	0.588	147.5 ± 103.8	105.2 ± 90.9	0.092
K-BNT	49.0 ± 5.1	49.3 ± 7.0	0.703	39.8 ± 9.2	45.2 ± 9.4	**0.021**
RCFT copy	32.1 ± 3.4	33.5 ± 2.2	0.344	24.8 ± 7.6	27.8 ± 7.5	0.112
SVLT immediate recall	21.2 ± 4.8	20.3 ± 5.4	0.913	15.1 ± 5.2	17.5 ± 4.9	0.056
SVLT delayed recall	6.7 ± 2.1	6.5 ± 2.8	0.956	3.5 ± 2.2	4.8 ± 2.8	0.054
SVLT recognition score	11 ± 0.9	10.6 ± 1.1	0.419	9.2 ± 2.2	9.8 ± 2.0	0.217
RCFT immediate recall	12.7 ± 5.6	16.4 ± 8.0	0.480	8.4 ± 5.5	12.2 ± 7.1	**0.018**
RCFT delayed recall	14.4 ± 5.3	15.5 ± 6.9	0.695	8.7 ± 5	12.1 ± 7.7	**0.042**
RCFT recognition score	10.7 ± 1.2	10.1 ± 1.1	0.340	9.5 ± 2.3	9.2 ± 2.7	0.680
Stroop color reading	70.2 ± 26.8	53.9 ± 27	0.276	45.8 ± 23.9	53.9 ± 24.9	0.181
COWAT-animal	15.5 ± 2.6	14.7 ± 2.9	0.507	10.5 ± 4.2	12.5 ± 3.7	**0.040**
COWAT-supermarket	20.7 ± 6.3	17.2 ± 5.6	0.276	9.9 ± 5.3	13.2 ± 5.9	**0.020**
COWAT phonemic	24.7 ± 11.1	21.8 ± 13.1	0.664	13.4 ± 8.6	16.2 ± 9.2	0.201

PSP, progressive supranuclear palsy; NC, normal cognition; MCI, mild cognitive impairment; SCC, subjective cognitive complaints; K-BNT, Korean version of the Boston Naming Test; RCFT, the Rey Osterrieth Complex Figure Test; SVLT, the Seoul Verbal Learning Test; COWAT, the Controlled Oral Word Association Test. Bold values represent statistically significant p-value (*p* < 0.05).

## Discussion

In the present study, we investigated SCC in patients with PSP. The results of the study indicated that SCC were commonly reported by the 83 PSP patients, with a prevalence of 43.4%, regardless of their underlying cognitive state (NC or MCI). When the patients were divided into NC and MCI groups, the prevalence of SCC was similar in the two groups. In the PSP patients with NC, there were no differences in clinical characteristics or neuropsychological test results between those who did or did not report SCC. However, in patients with PSP who had MCI, those who reported SCC exhibited more severe cognitive impairments in objective neuropsychological tests, especially in the domains of attention, executive function, language, and visual memory compared to PSP patients with MCI who did not report SCC.

As noted above, the overall prevalence of SCC in PSP patients without consideration of underlying cognitive state was 43.4%. The MCI − SCC group had the highest prevalence, followed by the MCI + SCC, NC − SCC, and NC + SCC groups. The prevalence of NC in the PSP patients (19.3%) was lower than the reported prevalence in two studies of PD patients (65.3% and 46.5%) (8, 11), resulting in a lower prevalence of NC + SCC (7.2%) in our PSP study population compared to studies of PD patients (28.1% and 12.1%) (8, 11). Additionally, the prevalence of MCI + SCC (36.1%) in our PSP study population was greater than that reported in PD patients (10.7% and 30.3%) (8, 11). Cognitive decline is prominent in the early stages of PSP and progresses more rapidly than PD (15). Therefore, PSP patients who report SCC may be less likely to retain NC and may progress to MCI or dementia even in the early stages of the disease.

Among the SCC reported by PSP patients, those related to the memory domain were the most common, while the executive/frontal cognitive domain was most frequently affected in objective tests (1, 3). We postulate two explanations for this discrepancy: PSP patients may experience difficulties identifying changes in executive/frontal function, and these challenges may be exacerbated by the memory or executive dysfunction that can reduce self-awareness (secondary unawareness) (24). Nonetheless, in this study, MCI + SCC patients performed worse on objective tests of cognition, suggesting that their complaints were not due to secondary unawareness. The study findings suggest that executive/frontal dysfunction may be more challenging to notice in daily life than memory complaints, indicating a need for more precise indicators to assess it (25). In addition, language dysfunction was also a frequently reported complaint among PSP patients with SCC and is commonly identified in objective neuropsychological testing of PSP patients (1).

In the present study, SCC were reported by 6 of 16 (37.5%) PSP patients with NC, which was lower than the reported median prevalence of SCC among PD patients with NC (48.8%) (7). Interestingly, there were no significant differences between the clinical and demographic characteristics, including depression (GDS), of the NC + SCC and NC − SCC groups in this study; in contrast, PD patients with NC + SCC reported more affective symptoms, such as depression, anxiety, and apathy, than those without SCC (6, 8, 26). The results suggest that NC + SCC in PSP patients is not induced by affective symptoms, but larger longitudinal studies are required to assess these inter-relationships.

The study found that 36.1% (30/83) of all PSP patients and 44.8% (30/67) of those with MCI were classified within the MCI + SCC group. There were no differences between the demographic or clinical characteristics of PSP + MCI patients who did or did not report SCC. However, the PSP patients with MCI + SCC had worse scores in the objective tests of cognition, particularly in attention (digit span forward and backward), language (K-BNT), visual memory (RCFT immediate recall and RCFT delayed recall), and fronto-executive (COWAT-animal and COWAT-supermarket) domains. The results suggest that presence of SCC in PSP patients with MCI might be associated with worse cognitive function, as has been reported for PD patients (14, 27). In PD, SCC is postulated to be a marker for progression of cognitive decline. Purri et al. conducted a 5 years follow-up study and found that patients who reported SCC at baseline were 2.61 times more likely to progress to MCI or dementia than those who did not report SCC (14). Similar results have been reported in longitudinal studies, where the presence of SCC at baseline was associated with a greater incidence of MCI at follow-up (11–13). Therefore, clinicians should be alert to the possibility of worsened cognitive dysfunction of progression to dementia in PSP patients who report SCC in order to provide timely diagnosis and treatment. However, further longitudinal studies of PSP patients are necessary to confirm this hypothesis. Finally, there are still no evidence-based treatments for PSP-cognitive impairment yet, but early assessment and therapeutic intervention of the cognitive domains focusing on attention, language, visual memory, and fronto-executive function may reduce the burden of cognitive impairment in PSP patients.

There were several limitations of the present study. First, we used semi-structured interviews to establish the presence of SCC because there is no associated validated tool in PSP-SCC. Various assessment tools for PD-SCC were introduced in previous studies, including simple memory-related questions, Subjective Cognitive Decline-Questionnaire (SCD-Q), Non-Motor Symptoms Scale Domain-5 (NMSs-5), Cognitive Complaints Interview (CCI), Subjective cognitive decline semi-structured interview, etc. (28). Patients with PD exhibit deficits mainly in executive function, attention and visuospatial function rather than memory and language. Similar to PD-SCC, we adopted our semi-structured interview including five domains to evaluate PSP-SCC at the same time. Second, there are no validated diagnostic criteria for MCI in PSP; however, as PD is the condition that has similar clinical symptoms with PSP making it acceptable to apply the diagnostic criteria of PD with MCI. Thirdly, we used the UPDRS and H&Y instead of PSPRS. As some important core features such as oculomotor and cognitive dysfunction would not be assessed by the UPDRS, further evaluation with videonystgmography and detailed neuropsychological tests was performed in any patients with a suspected diagnosis of PSP. Last, since this was a cross-sectional study, it is uncertain whether PSP patients with SCC had worse cognitive function compared to those who did not have SCC. A prospective longitudinal study is needed to address this question.

## Conclusion

The present study highlights the prevalence of SCC in PSP and its association with worse objective cognitive function in PSP patients with MCI. These findings suggest that SCC in PSP patients with MCI could be a worsening sign of cognitive function. Therefore, it is crucial for clinicians to assess SCC in PSP patients and to provide timely diagnosis and management of cognitive decline. These measures could lead to better outcomes for PSP patients with cognitive decline, ultimately improving their quality of life.

## Data availability statement

The raw data supporting the conclusions of this article will be made available by the authors, without undue reservation.

## Ethics statement

The studies involving humans were approved by the Institutional Review Board of Samsung Medical Centre. The studies were conducted in accordance with the local legislation and institutional requirements. The participants provided their written informed consent to participate in this study. Written informed consent was obtained from the individual(s) for the publication of any potentially identifiable images or data included in this article.

## Author contributions

JL: Conceptualization, Data curation, Formal analysis, Writing – original draft. JA: Conceptualization, Data curation, Formal analysis, Supervision, Writing – original draft, Writing – review & editing. JH: Conceptualization, Data curation, Writing – original draft. JY: Conceptualization, Data curation, Supervision, Writing – review & editing. JC: Conceptualization, Supervision, Writing – review & editing.
