# Supplementary material for: Subjective cognitive complaints in patients with progressive supranuclear palsy
Source: Front Neurol. 2023 Dec 14;14:1326571. doi: 10.3389/fneur.2023.1326571 (PMC10753004; doi:10.3389/fneur.2023.1326571)
Supplement: Supplementary file 1 [file Table_1.DOCX]

**Semi-structured interview to assess subjective cognitive complaints**

: The patients were asked to respond with either "yes" or "no." Those who reported problems in at least one cognitive domain in response to the following questions were classified as subjective cognitive complaints (SCC).

| **Cognitive domain** | **Questions** | **Answer** | **Cognitive tests** |
| --- | --- | --- | --- |
| Memory | ● Do you have a problem with your memory (e.g., forgetting medical appointments, forgetting where you put things, difficulty remembering conversations, difficulty remembering things that you want to do, or taking your medication at the right time)? | Yes/ No | SVLT  RCFT |
| Language | ● Do you have language problems (e.g., difficulty recalling the name of a word or object, difficulty comprehending the contents of news or movies, or problems with communication or conversing with others)? | Yes/ No | K-BNT |
| Visuospatial | ● Do you have visuospatial problems? (e.g., difficulty driving or using public transport, difficulty finding your way home, getting lost in the neighbourhood or familiar places)? | Yes/ No | RCFT |
| Attention | ● Do you have any problems with attention (e.g., difficulty concentrating on what you are doing, difficulty maintaining attention during a situation or activity, little things distract you from the task)? | Yes/ No | Digit span forward, Digit span backward, TMT-A, TMT-B |
| Executive/frontal function | ● Do you have any organizational difficulties, such as planning your day, or have you experienced personality change? (e.g., difficulty creating a schedule, difficulty managing finances, or exhibiting symptoms of apathy or impulsivity)? | Yes/ No | Stroop colour reading  COWAT |

SVLT, Seoul verbal learning test; RCFT, Rey Osterrieth complex figure test; K-BNT, Korean version of the Boston naming test; TMT-A, trail-making test type A; TMT-B, trail-making test type B; COWAT, controlled oral word association test.
